# sMICA/sMICB and Immune Checkpoint in Endometriosis: Toward a Minimally Invasive Diagnostic Model Based on Machine Learning

**DOI:** 10.3390/biomedicines14030647

**Published:** 2026-03-12

**Authors:** Anastasia Belevich, Maria Yarmolinskaya, Ilya Smirnov, Anastasia Stolbovaya, Olga Shashkova, Marina Samoylovich, Sergey Selkov, Polina Grebenkina, Elizaveta Tyshchuk, Dmitry Sokolov

**Affiliations:** Federal State Budgetary Scientific Institution, Research Institute of Obstetrics, Gynecology and Reproductology Named After D.O. Ott, 199034 St. Petersburg, Russia; m.yarmolinskaya@gmail.com (M.Y.); smirnov.iv.mail@gmail.com (I.S.); anastasia.stolbovaya@gmail.com (A.S.); ujinolga@yandex.ru (O.S.); mpsamoylovich@gmail.com (M.S.); selkovsa@mail.ru (S.S.); grebenkinap@gmail.com (P.G.); falcojugger@yandex.ru (D.S.)

**Keywords:** endometriosis, minimally invasive diagnosis, machine learning, MICA, MICB, endoglin, immune checkpoints

## Abstract

**Background:** Endometriosis is a complex condition that impairs women’s quality of life and reproductive potential. Its diagnosis remains significant challenge for clinicians. The aim of the study was to investigate cancer-like immune evasion mechanisms in endometriosis and to develop a novel diagnostic model using machine learning. **Methods:** In this study, we measured the levels of soluble forms of the following immune markers in blood serum and peritoneal fluid (PF): sMICA, sMICB, sEng, sCD25, s4-1BB, sB7.2, sCTLA-4, sPD-L1, sPD-1, sTIM-3, sLAG-3, and sGal-9. **Results:** sMICB levels in PF differed across endometriosis stages and were higher in patients with endometriosis-associated adhesions. sMICA levels in PF were elevated in women with endometriosis-associated infertility. The disease severity was inversely correlated with serum sB7.2 levels and positively correlated with serum sTIM-3 levels. A logistic regression model achieved an accuracy = 0.79, AUC = 0.94, and F1-score = 0.88, whereas XGBoost performed better with accuracy = 0.94, AUC = 0.95, and F1-score = 0.96. The key predictive features in both models were sMICB serum level and patients’ pain score. **Conclusions:** Our results demonstrate the potential role of sMICA and sMICB shedding in endometriosis and present a novel, minimally invasive diagnostic approach.

## 1. Introduction

Endometriosis is a prevalent gynecological disorder characterized by dysregulation of innate immunity, including impaired immune surveillance. In rare cases, endometriosis can also occur in extrapelvic locations, such as the lungs and the brain [1,2]. Despite being a benign gynecological disorder, endometriosis shares several features with malignant tumors, including local tissue invasion, neoangiogenesis, resistance to apoptosis, and evasion of immune surveillance [3]. The peritoneal microenvironment in endometriosis is characterized by a state of chronic inflammation, with altered function of immune cells such as natural killer (NK) cells, macrophages, and T lymphocytes [4,5]. This dysfunctional immune response fails to effectively clear ectopic endometrial cells, permitting their establishment and progression. Notably, this impairment in immune surveillance resembles the mechanisms employed by malignant cells to evade host immunity. Malignant cells often produce sMICA and sMICB, which downregulate NKG2D expression on NK and CD8^+^ T cells, thereby evading cytotoxicity [6]. Given that NK cell cytotoxicity is impaired in endometriosis—both in vitro and in vivo—it is plausible that sMICA and sMICB shedding by ectopic lesions represents a parallel immune evasion strategy [4]. Although the roles of NKG2D ligands and immune checkpoints are well established in oncology, their systematic evaluation in the context of endometriosis clinical heterogeneity (disease stage, adhesions, infertility) and their potential as serum biomarkers remain underexplored.

Another key mechanism by which cancer cells evade immune destruction is through the exploitation of immune checkpoints—inhibitory pathways, often mediated by ligand-receptor interactions (e.g., PD-1/PD-L1, CTLA-4/CD80/CD86). While these pathways are essential for maintaining self-tolerance and regulating immune responses, tumor cells can co-opt them to induce T-cell exhaustion and anergy [7]. Soluble forms of these checkpoint proteins can also modulate immune responses and have emerged as promising biomarkers in oncology [8,9,10]. Similar immune tolerance mechanisms may operate in endometriosis. For instance, ectopic endometrial cells have been shown to express PD-L1, potentially suppressing local immune responses in the peritoneal cavity and thereby promoting lesion survival [11]. Other checkpoint molecules such as TIM-3 and LAG-3—and their ligands, including galectin-3 and galectin-9—are associated with T-cell dysfunction in cancer and may similarly contribute to the immune-suppressive landscape of endometriosis [12,13,14]. While membrane-bound immune checkpoints (e.g., PD-L1) have been detected in endometriotic lesions [15], data on circulating soluble forms of these molecules—which could serve as systemic biomarkers—are extremely limited. In contrast, soluble checkpoints (sPD-1, sCTLA-4, sTIM-3) are already used in oncology for therapy monitoring and prognosis [16], strongly supporting their investigation in endometriosis.

Beyond direct immune checkpoint inhibition, other soluble factors are critical in shaping the peritoneal microenvironment. Elevated levels of sCD25—the alpha chain of the IL-2 receptor—can sequester IL-2 and serve as a marker of regulatory T cell activity, contributing to an immunosuppressive state [17]. Co-stimulatory molecules 4-1BB and B7.2 are essential for effective T cell activation, and their dysregulation may compromise the immune system’s ability to target endometriotic tissue [18,19]. TGF-β1, a potent immunosuppressive cytokine, promotes fibrosis—a hallmark of advanced endometriosis that is closely associated with chronic pelvic pain [20]. Endoglin (CD105), a co-receptor in the TGF-β signaling pathway, is upregulated on proliferating endothelial cells and is involved in angiogenesis, a process central to endometriosis progression [21]. Therefore, this study aimed to evaluate the levels of soluble immune checkpoints and mediators: sMICA (MHC class I Chain-related A), sMICB (MHC class I Chain-related B), sEng (Endoglin), sCD25, s4-1BB, sB7.2 (CD86/B7.2), sCTLA-4 (Cytotoxic T-Lymphocyte-Associated Protein 4), sPD-L1 (Programmed Death-Ligand 1), sPD-1, sTIM-3 (T cell Immunoglobulin and Mucin domain-containing protein 3), sLAG-3 (Lymphocyte Activation Gene-3), and sGal-9 (Galectin-9)—in the serum and PF of patients with endometriosis and endometriosis-negative controls. We sought to compare these levels across disease stages (revised ASRM stages I-IV, formerly known as the revised American Fertility Society (rAFS) and to investigate their potential correlations with clinical pain scores and the presence of peritoneal fibrosis [22]. Additionally, we developed a machine learning-based diagnostic model to predict endometriosis in women.

## 2. Materials and Methods

### 2.1. Study Participants and Sample Collection

The study included 66 women who underwent laparoscopic surgery and were diagnosed with endometriosis at the Department of Operative Gynecology of the D.O. Ott Research Institute of Obstetrics, Gynecology, and Reproductology. The inclusion criteria were: age 18–40 years, an intraoperatively confirmed peritoneal endometriosis verified by histological examination, and signed informed consent. Patients were divided into two groups: peritoneal endometriosis stages I–II (*n* = 18) and stages III–IV (*n* = 48) according to the ASRM classification. The control group (*n* = 16) included women who underwent diagnostic laparoscopy as part of their pre-IVF work-up due to male-factor infertility, in whom no endometriotic lesions or other gynecological pathology were identified.

The exclusion criteria for all groups were: decompensation of chronic somatic diseases, oncological diseases, acute infections or exacerbation of chronic infections, uterine fibroids, polycystic ovary syndrome, acute inflammatory pelvic diseases, autoimmune diseases, and the use of immunomodulatory or hormonal drugs within 3 months prior to surgery. The study was reviewed and approved by the Local Ethical Committee of the D.O. Ott Research Institute of Obstetrics, Gynecology, and Reproductology. Informed consent was obtained from all participants, allowing for subsequent anonymized processing and analysis of the results and their publication in open access. Study samples included peripheral blood collected during the early follicular phase of the menstrual cycle (days 5–12), prior to surgery, and peritoneal fluid obtained intraoperatively.

### 2.2. Assessment of Soluble Immune Checkpoint Protein Levels

The concentration of soluble immune checkpoint molecules (sCD25, s4-1BB, sB7.2, sCTLA-4, sPD-L1, sPD-1, sTIM-3, sLAG-3, sGal-9) in serum and PF was assessed using commercial kit (Cat No: 740962, BioLegend, San Diego, CA, USA) according to the manufacturer’s instructions. Samples (25 μL each) were premixed with 25 μL of assay buffer to achieve a two-fold dilution and transferred into wells of a 96-well plate. Capture beads specific to the target proteins were then added and incubated for 120 min. Following a wash step, detection antibodies were added to the wells and incubated for 60 min. Streptavidin-phycoerythrin solution was then added to the samples. After 30 min of incubation, the wells were centrifuged, washed, resuspended in assay buffer, and analyzed using a FACSCanto II flow cytometer (BD, San Jose, CA, USA). Each sample was analyzed in singlicate. The minimum detectable concentration is specified in the manufacturer’s instructions and varies for different analytes.

### 2.3. Assessment of sEng, sMICA, and sMICB Levels

Concentrations of sEng, sMICA, and sMICB were quantified using in-house ELISA assays with previously reported performance characteristics [23,24]. The limits of detection for sEng, sMICA, and sMICB were 0.232, 0.246, and 0.378 ng/mL, respectively. Intra-assay coefficients of variation were 5.0%, 4.4%, and 2.4%, while inter-assay coefficients of variation were 8.7%, 12.4%, and 20.8%, respectively. Commercial recombinant MICA and MICB proteins (R&D Systems, Minneapolis, MN, USA, cat. 1300-MA and 10431 MB, respectively) and recombinant endoglin (R&D Systems, Minneapolis, MN, USA, cat. 6578-EN) were used as calibrators for the in-house assays.

### 2.4. Statistical Analysis

Quantitative data are presented as median, lower (25%) and upper (75%) quartiles, arithmetic mean, standard deviation, standard error of the mean, 95% confidence interval for the mean, minimum, and maximum. Categorical data were described as frequencies and proportions with the calculation of the 95% confidence interval for the proportions. All quantitative data were initially tested for normality of distribution using the Shapiro–Wilk test, as well as tests for skewness and kurtosis, with calculation of the *p*-value for testing the null hypothesis of normal distribution. For data with a distribution close to normal, Welch’s *t*-test was used to compare two independent continuous variables; for data significantly deviating from a normal distribution, the Wilcoxon–Mann–Whitney test was used. For comparing three or more independent continuous variables with normal or near-normal distribution, one-way analysis of variance (one-way ANOVA) was used; for data with a non-normal distribution, the Kruskal–Wallis test was used. For multiple comparisons, the Holm-Bonferroni correction or the False Discovery Rate (FDR) correction was applied. Fisher’s exact test or Pearson’s chi-square test were used to analyze associations between categorical variables. Pearson’s or Spearman’s linear correlation was used to assess the relationship between biomarker levels in blood and peritoneal fluid, between biomarker levels and ASRM scores, and between biomarker levels and VAS pain scores. A *t*-test was used to assess the significance of the correlation, and a 95% confidence interval for r was calculated. Simple linear regression was used to quantitatively analyze the association between an independent variable (biomarker level) and a dependent variable (ASRM score), determining the direction and strength of the influence. The significance of the association was assessed using linear regression coefficients, their standard errors, 95% confidence intervals, and *p*-values from the *t*-test. Diagnostics of assumptions (assessment of normality of residuals, homoscedasticity) were performed to verify the appropriateness of using linear regression. The results were interpreted as the change in disease activity per unit change in the biomarker level, all other factors being equal. All reported *p*-values were based on two-sided tests of significance. Differences were considered statistically significant at *p* < 0.05. For multiple comparisons, the Holm–Bonferroni correction was applied. Statistical analysis was performed using the R programming language version 4.3.1 (R Foundation for Statistical Computing, Vienna, Austria).

### 2.5. Machine Learning Model Development and Validation

Due to the limited sample size, we employed a single stratified random split (seed = 42) into training (*n* = 49), validation (*n* = 16), and test (*n* = 17) sets, preserving the case–control ratio. All features included in the machine learning model (serum biomarkers and clinical variables) were complete for the entire cohort (*n* = 82); thus, no imputation was performed.

The XGBoost model used the following hyperparameters: objective = ‘binary:logistic’, eval_metric = ‘aucpr’, learning_rate = 0.15, max_depth = 4, subsample = 0.9, colsample_bytree = 0.7, with monotone constraints (VAS_pain_score ≥ 0, sMICB ≤ 0) based on biological plausibility. Training ran for up to 10,000 rounds with early stopping (patience = 150) monitored on the validation set.

Model performance was evaluated on the held-out test set. 95% CIs for AUC, accuracy, and F1-score were estimated via 1000 bootstrap resamples.

To assess the added value of biomarkers, we compared three models: (1) clinical variables only, (2) biomarkers only, and (3) combined. Feature contributions were interpreted using SHAP values (TreeExplainer) applied to the final XGBoost model on the independent test set (*n* = 17). SHAP values provide a game-theoretic measure of each feature’s marginal contribution to individual predictions, enabling both global and local interpretability.

## 3. Results

### 3.1. Comparison of sEng, sMICA, sMICB, and Checkpoint Protein Levels in Serum and PF

sEng, sMICA, and sMICB levels were measured in all 82 study participants, whereas soluble checkpoint proteins were assessed in 32 patients with endometriosis and 8 controls without endometriosis. Among these checkpoint proteins, no significant differences between the groups were observed for any analyte.

**Figure 1:**
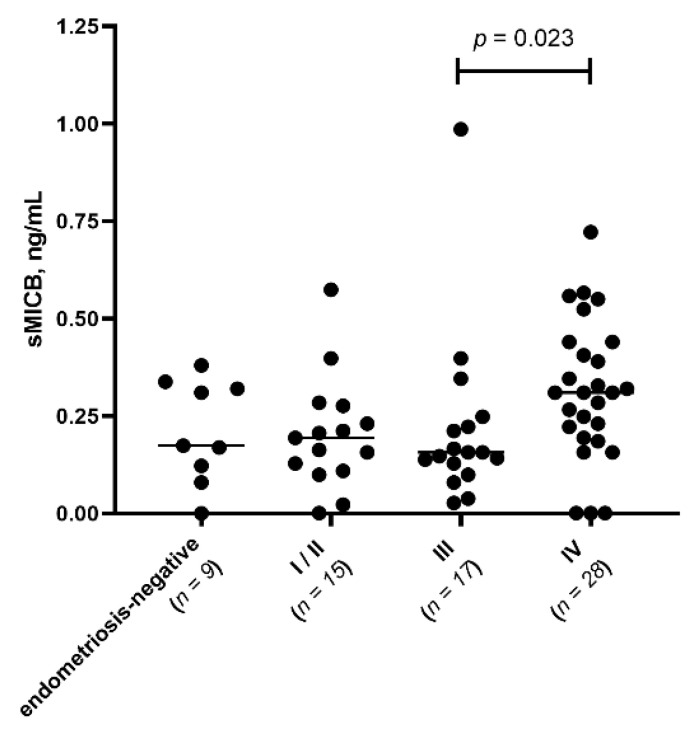
(Scatter plot). Comparison of sMICB median levels in PF between control group and patients with different stages of endometriosis. Control = 9; Stages I–II, *n* = 15; stage III, *n* = 17; stage IV, *n* = 26.

To assess the relationship between disease severity and soluble mediator profiles, we categorized patients with endometriosis into three groups according to rASRM disease stages. Total scores are used to categorize disease into four stages: Stage I (minimal; 1–5 points), characterized by few superficial implants and minimal or no adhesions; Stage II (mild; 6–15 points), with more numerous or slightly deeper implants and mild adhesions; Stage III (moderate; 16–40 points), featuring deep implants, ovarian endometriomas, and moderate adhesions; and Stage IV (severe; >40 points), reflects severe disease, which may include large endometriomas, dense adhesions, and/or deep infiltrating lesions. According to that classification we divided our patients into 3 groups: stages I–II, *n* = 18; stage III, *n* = 20; stage IV, *n* = 28. Among the analytes tested, sMICB levels in PF were significantly higher at stage IV compared to stage III (*p* = 0.023, Holm-adjusted *p* = 0.023) (Figure 1).

**Figure 2:**
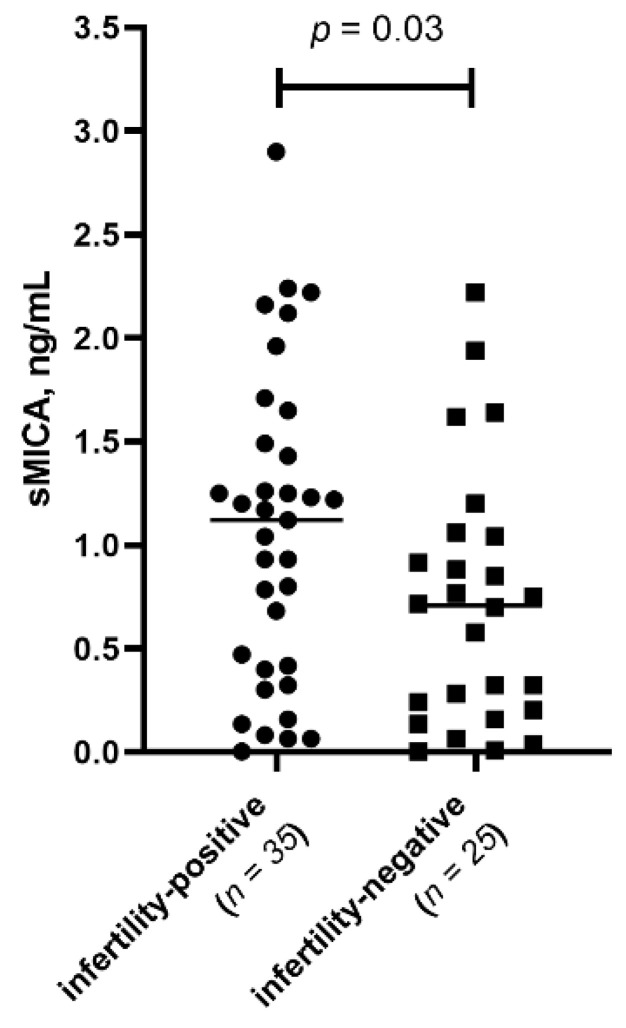
(Scatter plot). Comparison of sMICA median levels in PF between infertility-positive and infertility-negative endometriosis patients. Infertility-positive (*n* = 35); infertility-negative patients (*n* = 25).

We next evaluated whether infertility status influenced immune mediator levels, irrespective of endometriosis stage. Women were grouped as: patients with infertility (*n* = 35) and patients without infertility (*n* = 25). sMICA levels in PF were significantly elevated in women with endometriosis-associated infertility (*p* = 0.032) (Figure 2).

**Figure 3:**
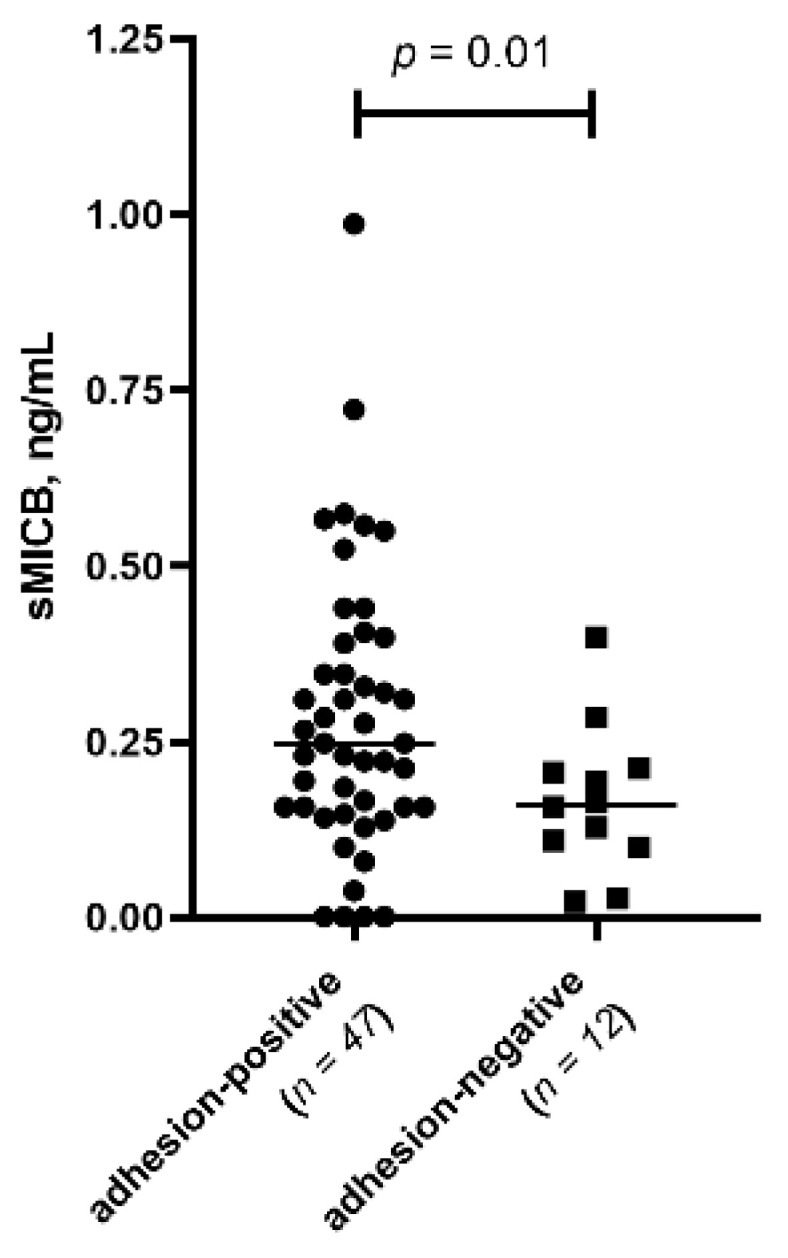
(Scatter plot). Comparison of sMICB median levels in the PF between adhesion-positive and adhesion-negative endometriosis patients. Adhesion-positive patients (*n* = 47); adhesion-negative patients (*n* = 12).

Given the clinical relevance of adhesions in endometriosis progression, we compared biomarker levels between patients with (*n* = 47) and without (*n* = 12) adhesions, irrespective of disease stage. sMICB levels in PF were higher in the adhesion-positive group (*p* = 0.01) (Figure 3).

### 3.2. Correlation Analysis

**Figure 4:**
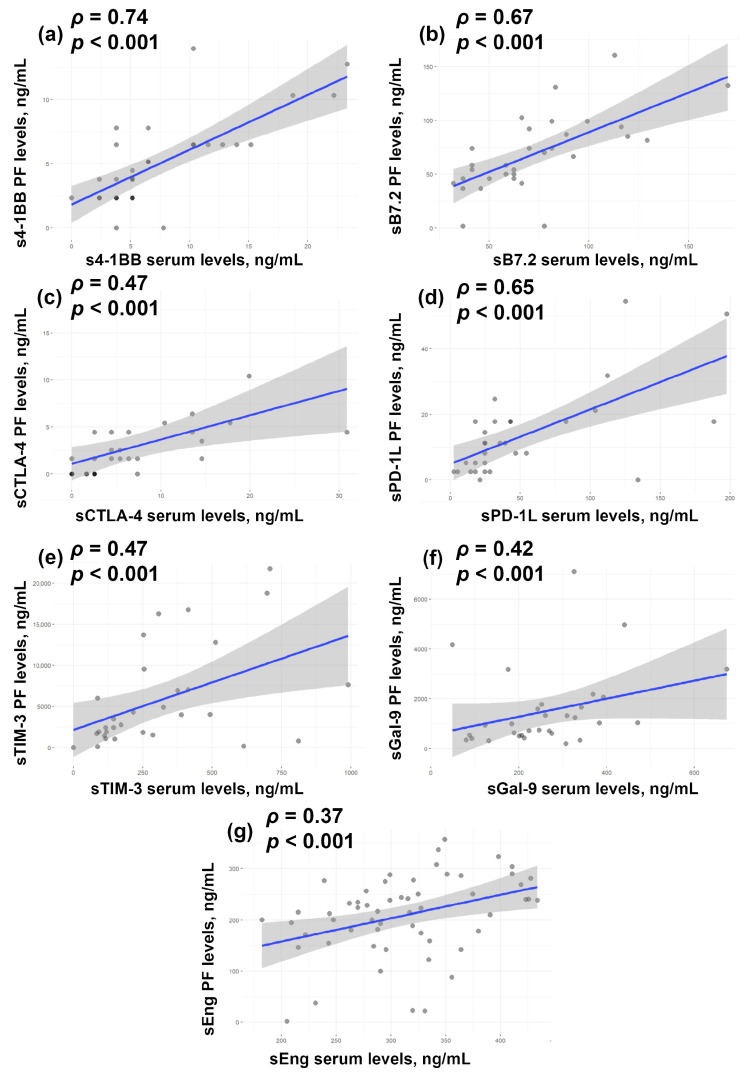
Spearman correlation between the levels of s4-1BB (**a**), sB7.2 (**b**), sCTLA-4 (**c**), sPD-L1 (**d**), sTIM-3 (**e**), sGal-9 PF (**f**), and sEng (**g**) in serum and PF. The blue line represents the linear regression line, and the grey shaded area indicates the 95% confidence interval.

In this study, we also evaluated correlations between protein levels in serum and PF in patients with endometriosis. Serum levels of s4-1BB (Figure 4a), sB7.2 (Figure 4b), sCTLA-4 (Figure 4c), and sPD-L1 (Figure 4d) showed a positive correlation with their levels in PF (*ρ* > 0.6, *p* < 0.001). A moderate correlation (*ρ* = 0.4–0.6, *p* < 0.001) was observed for sTIM-3 (Figure 4e), sGal-9 (Figure 4f), and sEng (Figure 4g).

**Figure 5:**
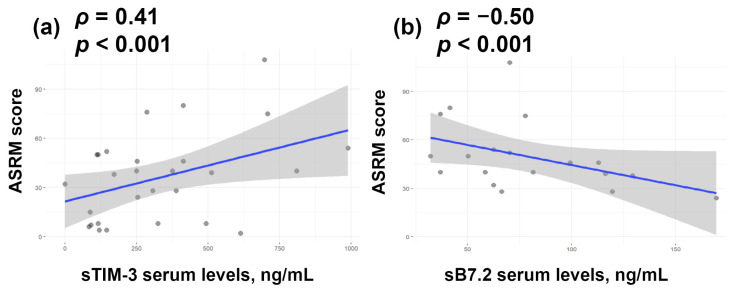
Spearman correlation between sTIM-3 (**a**) or sB7.2 (**b**) serum levels and disease severity assessed by ASRM scores. The blue line represents the linear regression line, and the grey shaded area indicates the 95% confidence interval.

We next assessed correlations between protein levels and pain severity (VAS) or disease stage (ASRM). No association with VAS was observed for any marker in either serum or PF. However, serum sB7.2 levels showed a moderate inverse correlation with disease stage, while serum sTIM-3 levels exhibited a direct correlation (*ρ* ≤ 0.5) (Figure 5).

### 3.3. Development of a Minimally Invasive Diagnostic Method for Endometriosis Using Machine Learning: Logistic Regression and XGBoost Gradient Boosting

In this study, we used two methods to predict the presence of endometriosis: logistic regression and the gradient boosting algorithm XGBoost. Logistic regression models the probability that a patient belongs to the target class (i.e., has endometriosis) as a function of a linear combination of independent variables (clinical, anamnestic, and laboratory-instrumental predictors). This is achieved through a logit transformation, which maps the linear predictor to a probability value between 0 and 1. The final model consists of coefficients optimized to minimize the loss function. Logistic regression is considered a benchmark method due to its high interpretability, which allows for a quantitative assessment of each factor’s contribution to the risk of developing the pathology. Gradient boosting is a fundamentally different approach that involves the sequential training of an ensemble of models, where each subsequent model specifically corrects the errors of the ensemble from previous iterations. The final model is a weighted sum of all constructed models, optimized to minimize a loss function. Key advantages of the algorithm are high predictive accuracy—often surpassing other methods—and robustness to outliers in the training data.

Since checkpoint protein concentrations were available for only 32 patients with endometriosis and 8 controls—a sample size too small for reliable comparison and model training—we used only serum levels of sEng, sMICA, and sMICB to develop the blood-based diagnostic algorithm. In addition, we included the following clinical variables: age, VAS pain scores, infertility status, and number of previous births. Model performance was evaluated using accuracy, F1-score, and ROC-AUC (area under the receiver operating characteristic curve).

**Figure 6:**
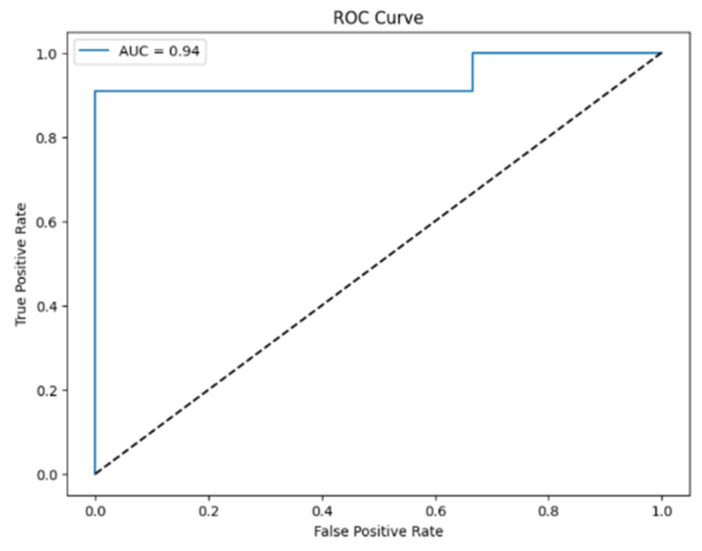
ROC (Receiver Operating Characteristic) curve for the logistic regression model. The dotted line represents the reference line for a random classifier (AUC = 0.5).

**Figure 7:**
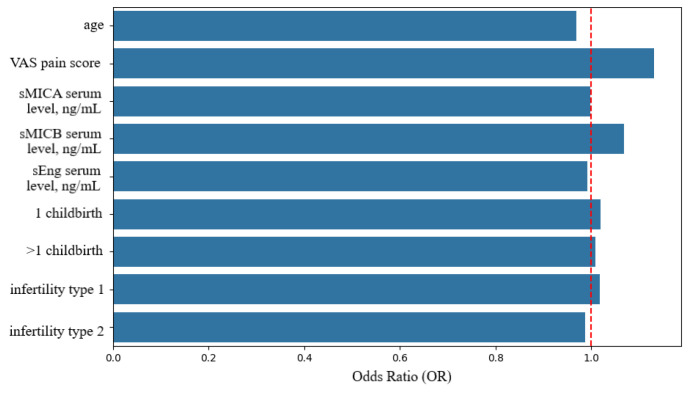
Contribution of clinical and biomarker variables to endometriosis risk prediction in the logistic regression model. VAS—Visual Analogue pain Score. The red dotted vertical line represents the reference value (1.0). Bars extending beyond this line indicate variables with significant predictive value.

The logistic regression model achieved an accuracy of 0.79, indicating a moderate ability to classify patients as either endometriosis-negative or having endometriosis. An AUC of 0.94 and an F1-score of 0.88 were obtained (Figure 6). VAS pain scores and serum sMICB levels were the strongest predictors of endometriosis (Figure 7).

**Figure 8:**
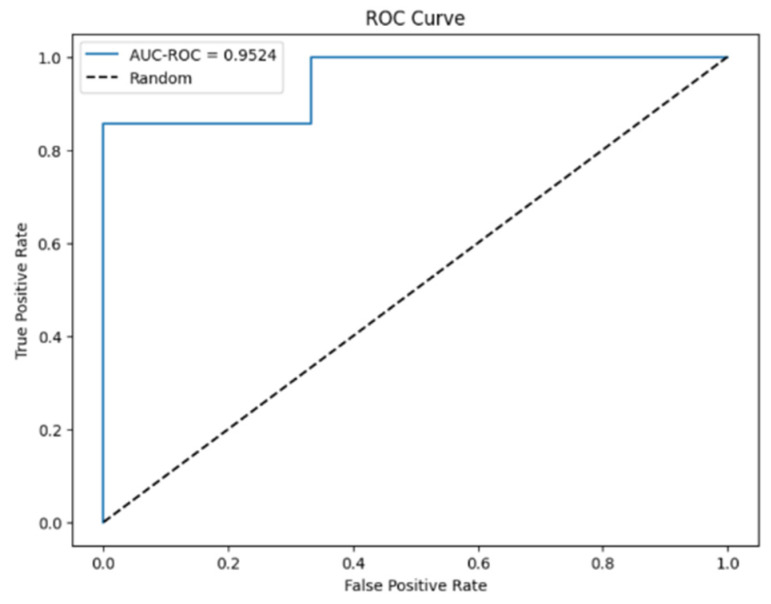
ROC curve for the XGBoost gradient boosting model. The dotted line represents the reference line for a random classifier (AUC = 0.5).

**Figure 9:**
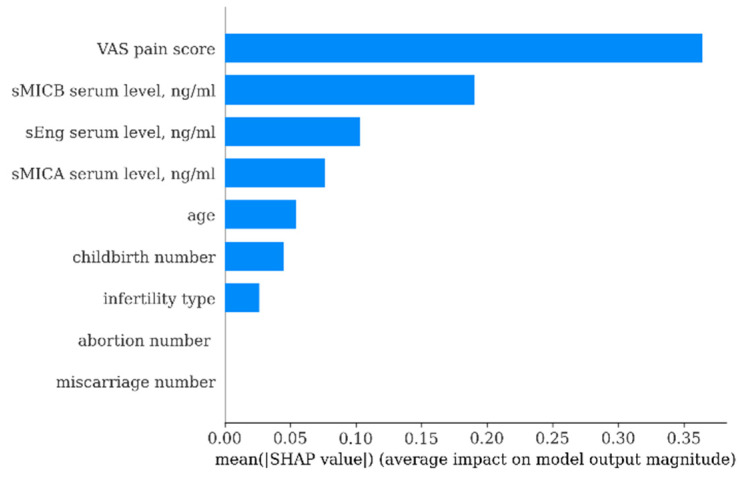
SHAP Summary Plot (SHapley Additive exPlanations) for the XGBoost model. VAS—Visual Analogue pain Score.

The predictive model created using XGBoost showed higher performance: accuracy = 0.94, AUC = 0.95, F1-score = 0.96 (Figure 8). As shown in Figure 9, the most significant predictors in this model were also serum sMICB levels and VAS pain scores.

## 4. Discussion

In our study, we observed statistically significant differences in sMICB levels in PF across endometriosis stages, with the highest levels detected in stage IV. Moreover, elevated sMICB levels were associated with the presence of peritoneal fibrosis. Furthermore, among patients with peritoneal fibrosis, sMICA levels were significantly higher in those with concurrent endometriosis-associated infertility. These findings are consistent with previous reports. The first study to investigate sMICA/B concentrations in PF of women with endometriosis was published in 2015. The authors reported elevated levels of soluble NKG2D ligands (particularly sMICA and sMICB) in PF, especially in severe cases [25]. Subsequent studies also found increased sMICA and sMICB levels in PF from patients with deep infiltrating endometriosis [26,27]. This elevation may represent a pathogenic mechanism by which endometriotic cells evade immune surveillance, analogous to the strategy employed by cancer cells.

Our finding that sMICB levels in peritoneal fluid are significantly elevated in patients with peritoneal fibrosis aligns with the growing body of evidence implicating immune–stromal crosstalk in endometriosis progression. In this context, recent work by Warzecha et al. demonstrated that extracellular matrix (ECM) remodeling markers—fibronectin and collagen IV—are markedly increased in both peritoneal fluid and plasma of women with endometriosis, with the highest concentrations observed in stage IV disease and in cases with dense adhesions [28]. Notably, peritoneal fluid fibronectin showed strong diagnostic performance (AUC = 0.94) and correlated with disease severity, reinforcing the concept that local tissue remodeling and fibrosis are integral to advanced endometriosis pathology. While our study focuses on soluble immune mediators, the convergence of findings—elevated sMICB, fibronectin, and collagen IV in fibrotic endometriosis—suggests a coordinated interplay between immune evasion and ECM dysregulation. Future studies integrating immune checkpoints with stromal biomarkers may further refine phenotypic stratification and improve non-invasive diagnosis.

Our study is the first to evaluate correlations among multiple soluble immune checkpoint proteins in patients with endometriosis. s4-1BB, sB7.2, sCTLA-4, sPD-L1, sTIM-3, sGal-9, and sEng levels correlated between serum and PF, indicating that these checkpoints may derive from the peritoneal cavity. Serum sB7.2 levels showed an inverse correlation with endometriosis stage (ASRM score). Given that the B7.2–CTLA-4 interaction inhibits T-cell activation, we hypothesize that elevated soluble B7.2 may reflect increased engagement with its inhibitory receptor, potentially contributing to T-cell dysfunction in advanced disease [29]. Similarly, the positive correlation between serum sTIM-3 and ASRM score aligns with a recent report of reduced TIM-3^+^ T cells in peripheral blood of women with endometriosis [12]. It is plausible that TIM-3 shedding from T cells—leading to decreased surface expression and increased soluble levels—may represent one mechanism of immune dysregulation in endometriosis [30]. The analysis of soluble immune checkpoint proteins was restricted to a subset of 40 participants. While we observed significant correlations between serum and peritoneal fluid levels for several checkpoints, these findings require validation in larger cohorts and should be interpreted as preliminary.

A key limitation of our study is the absence of a pre-study power calculation. The sample size was determined based on clinical feasibility and recruitment capacity at a single tertiary center, rather than on a priori statistical power estimation. While our pilot cohort enabled the detection of several statistically significant associations—particularly for sMICB and sMICA in relation to disease stage, adhesions, and infertility—the limited number of participants increases the risk of both Type II errors (false negatives) and overestimation of effect sizes. Consequently, our findings should be interpreted as hypothesis-generating rather than definitive. Future multicenter studies with larger, prospectively powered cohorts are needed to validate these results and assess the clinical utility of the proposed diagnostic model.

Another limitation is the nature of our control group: although all control participants were confirmed to be free of endometriosis via diagnostic laparoscopy—the current gold standard for exclusion of peritoneal disease—they were recruited from a population undergoing pre-IVF evaluation due to male-factor infertility. While this ensures the absence of endometriosis, it may introduce subtle confounding factors (e.g., hormonal, psychological, or inflammatory changes related to infertility).

In recent years, artificial intelligence and machine learning have seen expanded use in medicine, including development of novel non-invasive methods for diagnosing endometriosis. These approaches can integrate a wide spectrum of biomarkers to estimate the likelihood of disease. Several machine learning studies exploring factors such as miRNAs, serum inflammatory markers, and cancer biomarkers (e.g., CA-125) have demonstrated promising diagnostic potential [31,32,33,34]. In addition to serum biomarkers, genomic profiles, and imaging data, some studies enhance the performance of machine learning algorithms by integrating these modalities with key clinical features of endometriosis [35].

Although the strongest phenotype-specific associations were observed in peritoneal fluid—where sMICA linked to infertility and sMICB to adhesions and disease stage—we deliberately developed our diagnostic model using serum biomarkers due to the clinical impracticality of routine peritoneal fluid sampling. This decision is further supported by emerging evidence that systemic biomarkers may better reflect clinical manifestations than local pelvic concentrations. Notably, Bartnik et al. found no association between peritoneal fluid levels of ZEB1/ZEB2 and either endometriosis diagnosis or infertility, whereas plasma ZEB2 was significantly elevated in infertile women [36]. This demonstrates that circulating biomarkers can capture clinically relevant pathways—such as epithelial–mesenchymal transition dysregulation—that are not evident in the local microenvironment. Similarly, in our cohort, serum sMICB and sMICA showed significant correlations with their peritoneal counterparts and, when combined with clinical variables (notably VAS pain score), enabled high-performance prediction of endometriosis. Thus, while peritoneal fluid provides superior biological resolution, serum offers a clinically feasible and surprisingly informative surrogate for minimally invasive risk stratification.

Leveraging these insights, we developed a minimally invasive diagnostic model combining serum sMICB and pain scores with machine learning: the XGBoost algorithm achieved high performance (accuracy = 0.94, AUC = 0.95, F1-score = 0.96), outperforming logistic regression. However, it is critical to emphasize that this model was developed and evaluated on a single-center cohort and has not undergone external validation. Given the risk of overfitting, independent validation in a prospective, multi-center cohort is essential before any clinical application. This represents the primary limitation of our current machine learning approach and the most urgent next step for translation. Moreover, the clinical utility and calibration of the diagnostic model were not assessed due to cohort size constraints. Future studies should evaluate whether the model improves diagnostic decision-making compared to current practice (e.g., reducing unnecessary laparoscopies).

## 5. Conclusions

Our study provides evidence that soluble NKG2D ligands—particularly sMICB and sMICA—are differentially expressed in the peritoneal microenvironment of women with endometriosis and are associated with distinct clinical phenotypes of the disease. We demonstrate that sMICB levels in peritoneal fluid increase with disease severity, reaching the highest concentrations in stage IV endometriosis, and are significantly elevated in patients with endometriosis-associated adhesions. In contrast, sMICA is specifically linked to endometriosis-related infertility, suggesting an association with different clinical manifestations of the disease.

Importantly, we observed significant correlations between peritoneal fluid and serum levels of multiple immune checkpoint molecules—including sMICB, sMICA, sTIM-3, sB7.2, sCTLA-4, and sPD-L1—indicating that systemic circulation reflects local immune dysregulation in the pelvic cavity. This supports the potential use of serum-based biomarkers as indicators of disease activity and phenotype.

Building on these findings, we developed a minimally invasive diagnostic model combining serum levels of sMICB, sMICA, and sEng with key clinical variables (dysmenorrhea severity, infertility status, age, and parity). Both logistic regression and XGBoost models achieved high performance (AUC > 0.94), with serum sMICB and pain score emerging as the strongest predictors. Given the absence of clinically validated non-invasive tools for endometriosis diagnosis, this approach represents a promising step toward early detection and personalized management.

## Data Availability

The original contributions presented in this study are included in the article. Further inquiries can be directed to the corresponding authors.
